# A numerical analysis of skin–PPE interaction to prevent facial tissue injury

**DOI:** 10.1038/s41598-021-95861-3

**Published:** 2021-08-10

**Authors:** Rikeen D. Jobanputra, Jack Hayes, Sravani Royyuru, Marc A. Masen

**Affiliations:** Tribology Group, Department of Mechanical Engineering, Imperial College London, London, UK

**Keywords:** Biomedical engineering, Mechanical engineering, Occupational health, Public health, Computational methods

## Abstract

The use of close-fitting PPE is essential to prevent exposure to dispersed airborne matter, including the COVID-19 virus. The current pandemic has increased pressure on healthcare systems around the world, leading to medical professionals using high-grade PPE for prolonged durations, resulting in device-induced skin injuries. This study focuses on computationally improving the interaction between skin and PPE to reduce the likelihood of discomfort and tissue damage. A finite element model is developed to simulate the movement of PPE against the face during day-to-day tasks. Due to limited available data on skin characteristics and how these vary interpersonally between sexes, races and ages, the main objective of this study was to establish the effects and trends that mask modifications have on the resulting subsurface strain energy density distribution in the skin. These modifications include the material, geometric and interfacial properties. Overall, the results show that skin injury can be reduced by using softer mask materials, whilst friction against the skin should be minimised, e.g. through use of micro-textures, humidity control and topical creams. Furthermore, the contact area between the mask and skin should be maximised, whilst the use of soft materials with incompressible behaviour (e.g. many elastomers) should be avoided.

## Introduction

During the COVID-19 pandemic, healthcare professionals globally have been using personal protective equipment (PPE) for increased durations. The prolonged use of facial PPE, such as respirator masks, visors and face shields, may lead to the development of a range of skin issues, including irritation and injuries such as skin tears, pressure injuries and urticaria^1–6^. Respiratory protective equipment has been widely reported to cause skin reactions such as contact dermatitis, acne, facial itch and rash^7–9^. Lan et al. report skin damage related to general protective measures occurring in up to 97% of health care workers, of which the nasal bridge has the highest prevalence^10^. Yen et al. report that up to 71% of healthcare workers who wear PPE experience burning and itching sensations^11^. Discomfort and irritation may lead to the improper use of PPE, whilst skin injury might result in lost hours with medical and care staff being absent from work. In addition, a compromised skin barrier adds a potential entry route for COVID-19 infection^12^. Jiang et al. correlated PPE-induced skin injury with heavy sweating, the use of higher-grades of PPE and the duration of continued use of PPE^13^.

A key feature of tight-fitting or sealing PPE is that there is a close contact between the PPE and the skin, resulting in the skin being subjected to a combination of normal and shear forces. Manifestations of these loads acting on the skin range from indentation marks at the locations of PPE-skin contact to deep-tissue bruising across a larger area. Excessive loading of the skin can result in lesions at the skin surface, which can develop into erythema and mild irritations ^12,14–16^^.^ Various causative pathways to severe skin injuries have been presented in literature^17^. Loading of the skin may result in occlusion of the capillaries and restricted lymph flow, which will set off a cascade of biochemical processes. The resulting ischaemic response of the cell includes hypoxia, lack of nutrients and the build-up of metabolic waste products and will lead to a breakdown of cell organelles, triggering apoptosis or necrosis^18–20^. These effects at the cellular level, caused by applied external forces and local tissue deformation, result in macroscopic tissue injury at the sites of bony prominences, such as the nasal bridge, cheekbones and forehead^13,21^.

It has long been established that shear forces acting on the skin result in damage occurring at significantly lower pressures than when only a normal load is applied^22–25^ . In the contact between skin and PPE, three primary mechanisms can be identified that generate shear stresses at the skin interface. Firstly, static friction, sometimes also referred to as ‘shear’ or ‘stiction’, which prevents sliding of the PPE. Secondly, local relative motion between the PPE and skin, e.g. as a result of speaking when wearing PPE, causing rubbing of the PPE against the skin. Finally, upon compression, shear forces will develop at the interface^23–25^, due to the mask moving relative to the skin as well as the skin and PPE deforming perpendicular to the direction of loading by different amounts due to their respective Poisson’s ratios.

Preventing PPE-related skin injury requires a better understanding of the interaction between the PPE and the skin, in addition to the effects of this interaction on the strains and stresses inside the tissue. Common treatments to alleviate friction-related injuries involve the application of hydrocolloid dressings ^26,27^ and the use of moisturisers. However, it was found that incorrect applications of moisturisers before and after PPE application may increase infection risk^28^. Previous investigations on PPE have mainly focussed on modelling the pressure that acts on the surface of the skin, with the objective of ensuring an appropriate seal and maintaining a level of user comfort^29–31^. However, the effects of PPE-skin interaction on the stresses and strains inside the tissue have not previously been investigated. Finite Element Analysis (FEA) is an efficient tool to model and visualise the local subsurface stress and strain levels within the tissue ^32^ and will be used to provide insight into the effects on the skin of interacting with PPE.

The purpose of this investigation is to understand how the mechanical burden on the skin is affected by the characteristics of the PPE in terms of its material, geometric and interfacial properties. To enable this, the interaction between skin and a model respirator mask is examined using a parameterised finite element model. Following Oomens’ work on tissue damage^33^, the strain energy density (SED) in the skin was taken as the quantitative measure representing local tissue failure. A series of simulations enabled the quantification of the efficacy of the respirator mask alterations and its interaction with facial skin, in order to provide information on how to reduce skin injury amongst PPE wearers. A set of readily implementable guidelines regarding the use and design improvements of PPE will be defined based on the obtained results.

## Results

The parametric study was conducted using FEA to investigate the effects of independently altering the mask material, and its geometric and interfacial properties. The mask is loaded against the skin with a load of 5 N and subsequently subjected to a lateral motion of 2 mm to represent the sliding and relative motion of tightened/fitted PPE during day-to-day tasks, thus inflicting shear stresses on the skin. The facial injuries observed in users of PPE are representative of a combination of deep-tissue injury, such as bruising and more superficial injuries, such as blistering and abrasion. Using a combination of in-vivo experiments and finite element modelling, Oomens^33^ related the extent of localised tissue breakdown to the amount of deformation energy at that site. This metric was therefore adopted in the present study, recording the maximum SED in the dermis during contact with the mask. Material characteristics that were varied within the study were the Young’s modulus $E$ and the Poisson’s ratio $\nu$ of the respirator mask. The geometric and interfacial properties altered in the investigation respectively include the contact length, ${L}_{c}$, representative of the contact area between the skin and PPE, and the adhesive friction coefficient, $\mu$. Whilst the material and the geometry can be directly controlled by the designer, the friction in the contact is affected by a wide range of parameters, including the material being used and the surface microgeometry or texture, personal traits such perspiration and hairiness, as well as the application topical creams or lubricants. Table 1 lists the reference values used in this work, as well as the range of values investigated^29–31^.

**Table 1:** The mask material, geometry and interfacial properties that were varied in this study.

Parameter	Reference value	Variations in study
Applied load (N)	5.0	–
Elastic modulus (kPa)	100	2–10,000
Poisson's ratio (–)	0.4	−0.2 to 0.49
Length of skin-PPE contact (mm)	4.0	3.0–4.8
Coefficient of friction (–)	0.5	0.1–1.1

**Figure 1:**
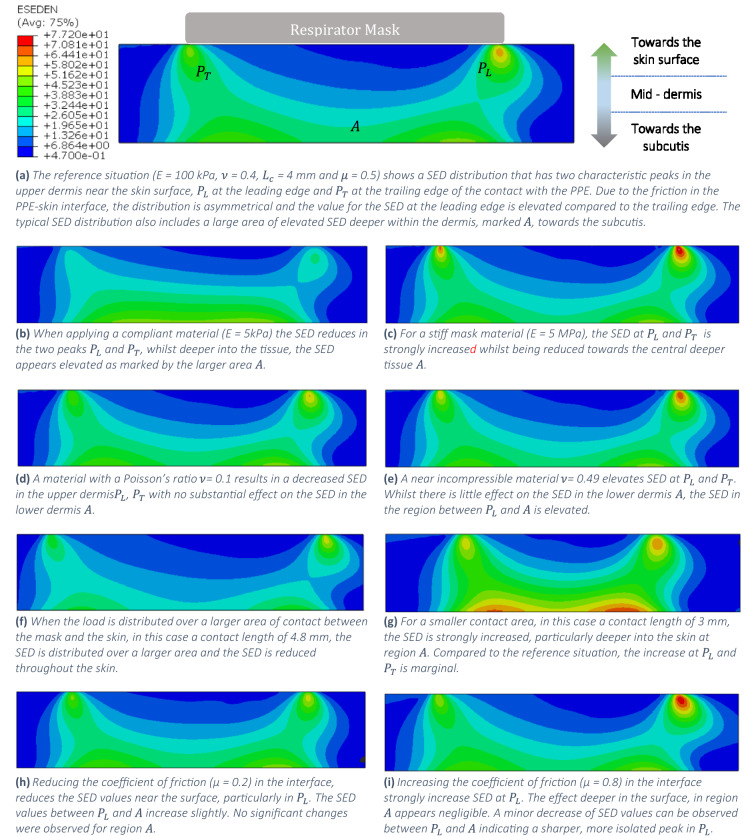
Close-up of the contact, showing the distribution of the SED in the dermis as a result of using PPE. Red indicates an elevated value. Distribution displayed overlayed over the undeformed tissue. Note that these images only show a portion of the skin, the used finite element model is larger, as shown in Fig. 5.

Figure 1a displays the SED distribution in the dermis resulting from the contact with PPE for the reference mask indicated in Table 1. These images display only part of the entire model, which is shown in Fig. 5. In all simulated cases the interface was fully closed, and there was no remaining gap between skin and PPE. The SED distribution has a characteristic shape, with three main regions of interest:

- a sharp peak, ${P}_{L}$, in the upper dermis close to the skin surface, at the leading edge
- a sharp peak, ${P}_{T}$, in the upper dermis close to the skin surface, at the trailing edge,
- a larger region, $A$, of elevated SED values deeper into the tissue close to the boundary between the dermis and the hypodermis.

Figure 1b–i illustrate the effects on the dermal SED distribution of varying the mask material, geometry and interfacial properties from the reference case. The various graphs show that increasing the stiffness of the material, the use of incompressible materials, and materials with a large coefficient of friction against skin all strongly increase the SED values, but mainly near the skin surface. Reducing the area of contact between the respirator mask and the skin strongly increases the SED deeper in the skin. From these results it can be concluded that to reduce the SED in the skin requires investigating a combination of effects. In terms of the design of optimised PPE, the stiffness or modulus of the mask material is arguably the main design parameter. A wide range of materials are available for mask design, and the stiffness directly affects the contact area and contact pressure, as well as the friction in the skin-PPE interface. Therefore, the results obtained in this study will be presented as a function of the modulus of the mask material.

### Stiffness of the mask material

**Figure 2:**
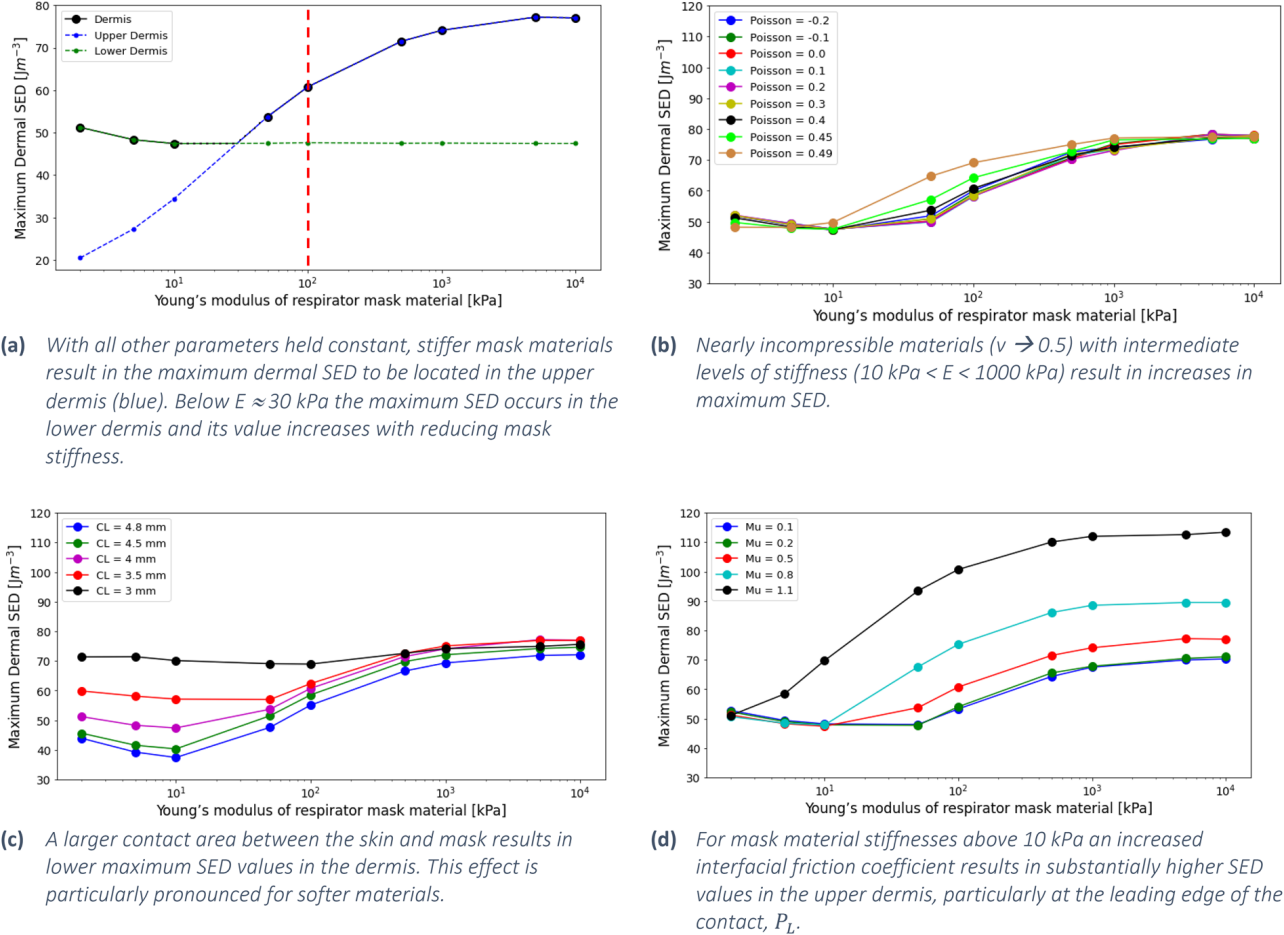
The effects of independently altering the mask properties on the maximum SED in the tissue. **(a)** Mask material stiffness, **(b)** Poisson's ratio, **(b)** area of contact, and **(d)** interfacial friction coefficient.

The effect of altering the stiffness of the mask material is clearly visible in all four graphs in Fig. 2, which show the evolution of the maximum dermal SED as a function of the stiffness of the mask material for a variety of cases. Taking Fig. 2a, which illustrates changes to the stiffness of the reference masks stiffness, the maximum SED in the skin increases following an S-shaped trend with increasing mask material stiffness. In general, a reduction of the mask stiffness leads to reduced maximum dermal SED in the upper dermis, as represented by the blue coloured curve. The SED in the lower dermis is not sensitive to the stiffness of the mask material, except for highly compliant materials, $E<10 {\text{kPa}}$, which result in a slight increase in SED (green curve in Fig. 2a). Figure 2b–d, show that the SED appears have a minimum level for compliant mask materials ($E\le 50 {\text{kPa}}$), whilst the SED plateaus at a maximum value for stiff mask materials with $E\ge {10}^{3} {\text{kPa}}$.

### Poisson’s ratio

The Poisson’s ratio describes the extent of deformation of a material perpendicular to the direction of loading, in this case the deformation of the mask material parallel to the skin surface when compressed against the skin. If the resulting deformation of the material is different than the deformation of the skin, an additional shear component is introduced in the interface. Therefore, the Poisson’s ratio of the mask material is a potentially interesting parameter to take into account during the design phase of respirators. Figure 2b illustrates that in general the effect of changing the Poisson’s ratio on the SED in the skin is small and may be ignored. However, for mask materials with an intermediate stiffness $(10 \mathrm{kPa}<E<500 \mathrm{kPa})$ the near incompressibility of rubber materials ($\upnu =0.49$) may result in an increase of the SED. For example, for a mask with a Young’s modulus of 50 kPa, this increase is more than 20%, from $\Xi =53.7 \text{J }{\text{m}}^{3}$ when $\upnu =0.40$ to $\Xi =64.8 \text{J }{\text{m}}^{3}$ when $\upnu =0.49$.

### Size of the contact interface

**Figure 3:**
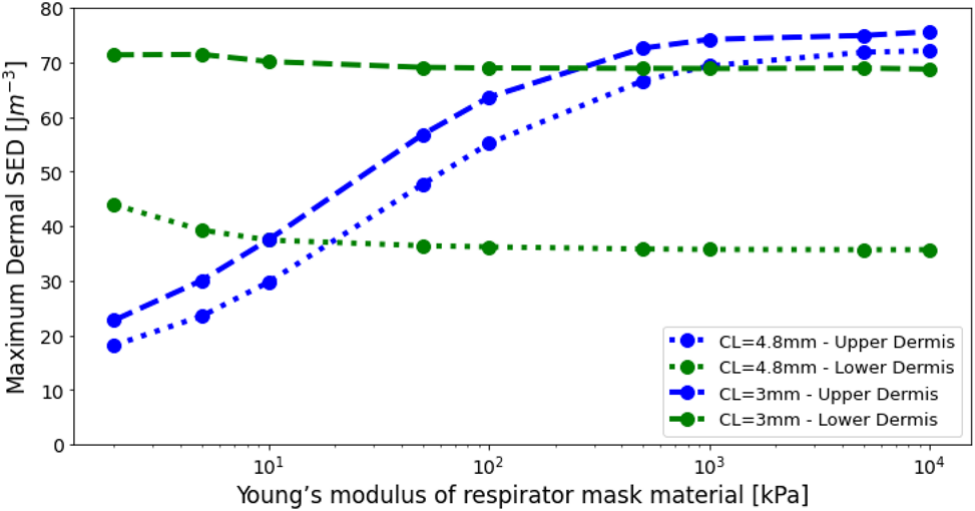
Evolution of SED in the tissue as a function of mask material modulus for contact lengths of 4.8 mm and 3 mm. Changes in contact length had a substantial effect on maximum SED in the lower dermis compared to the upper dermis.

Figure 2c shows that an increase of the contact area between the skin and the PPE results in decreased maximum SED values. For very soft materials this effect is quite pronounced; for $E=10 {\text{kPa}}$ the maximum SED reduces 47%, from $\Xi =70.1 \text{J }{\text{m}}^{3}$ for a contact length $L=3\text{ mm}$, to $\Xi =37.5 \text{J }{\text{m}}^{3}$ for a contact length $L=4.8\text{ mm}$. For stiff mask materials ($E>500\text{ kPa}$) the SED and approach a value of about $\Xi \sim 70 \text{J }{\text{m}}^{3}$, irrespective of the contact length. For very small contact sizes ($L\sim 3\text{ mm}$) the maximum value of the SED in the skin appears to be relatively insensitive to the stiffness of the mask material, with the curve being nearly horizontal. It was found that increases in contact significantly reduced SED values in region $A$, whilst having a much smaller effect on the upper dermis (Fig. 3).

### Coefficient of friction in the skin–PPE interface

Figure 2d summarises the effects of the interfacial friction on the SED in the skin. The coefficient of friction has a large effect on maximum dermal SED, compare Fig. 1h,j which represent µ = 0.2 and 0.8, respectively. Elevated friction increases the maximum SED in the upper dermis, particularly at location ${P}_{L}$, with little to no effect on the values in the lower dermis. At elevated levels of friction, the value of the SED increases more with increasing friction. Overall, lower friction values can lead to a strong reduction of the SED in the dermis. There appears to be a minimum friction level of approximately $\mu =0.2$, where a further reduction does not substantially maximum dermal SED.

## Discussion

The presented results provide a general overview of the relationships between the characteristics of the respirator masks and the resulting burden on the skin as quantified using SED. The dermal SED distributions provide some insights into the underlying tissue damage mechanisms, and thus can be used to develop PPE design guidelines to reduce the likelihood of dermal injury. With this in mind, it is worth noting that whilst in this work the SED was used as the indicator for tissue damage and has been related to the onset of tissue injury under sustained loading^33^, similar trends were obtained when analysing engineering parameters such as shear stress and deviatoric stress.

### Limiting the stressing of the skin

Figure 1 displays a range of dermal SED distributions in the skin as a result of combined compression and translation of the mask against the face. Elevated SED values in the upper dermis towards the skin surface may indicate an increased risk of superficial skin injury, such as abrasions, surface rupture and tearing as well as delamination of the dermal–epidermal junction. Elevated values of the SED deeper in the skin may indicate an increased likelihood of deep tissue injuries such as bruising, full skin rupture and delamination of the skin from the underlying tissue.

### Contact area

The effect the area of contact between the PPE and the skin is clearly visible when comparing Fig. 1a,f,g. Reducing the contact area means the load is distributed over a smaller area, meaning the SED is strongly increased throughout the skin, and particularly deeper in the skin at the region marked $A$ (Figs. 1g and 3). These results illustrate the importance of distributing the loads over a larger area, and thus the risk of pressure related injuries.

### Stiffness

Materials with a reduced Young’s modulus, often referred to as ‘softer’ materials, deform more under the same load than stiffer materials. As a result, the area of contact between the PPE and the skin increases and the strapping force of the PPE is distributed over a larger area. This supports the convention of softer materials being used in PPE with the aim of reducing discomfort and preventing injury. An additional effect of the increased area of contact between skin and PPE is that the maximum shear stress moves deeper into the tissue^34^. In Fig. 2a it can be seen that reducing the stiffness of the PPE from 10 to 2 kPa results in a modest but noticeable increase of the SED in the skin, driven by the lower dermis. When the stiffness of the mask material is larger than 1 MPa, i.e. significantly stiffer than the dermis, it was found that the maximum SED in the dermis did not vary significantly with further increasing stiffness. In that case the contact behaviour is dominated by the deformation of the skin whilst the PPE does not significantly deform.

### Poisson’s ratio

The Poisson’s ratio of the mask material quantifies the extent to which the material displays the Poisson effect, i.e. its deformation in lateral direction following compression of the mask material against the skin. Theoretically, values for the Poisson’s ratio range from $-1<\mathrm{v}<0.50$ and typical engineering materials have a Poisson’s ratio of approximately $\upnu =0.3$. Some cork materials do not display Poisson’s effect-like behaviour and thus have $\upnu =0$. Most rubber materials have a value of $\upnu \to 0.50.$ This means that rubber, whilst highly deformable, has a constant volume which does not change when loaded or pressurised. Therefore, materials with $\upnu =0.5$ are referred to as incompressible. If the lateral deformation is different from the lateral deformation of the skin, a shear stress may be generated in the interface. The results indicate that the SED is relatively insensitive to this phenomenon, except for the specific combination of the mask comprising a material with intermediate stiffness ($10 {\text{kPa}}<E<500 {\text{kPa}}$) and near incompressible behaviour ($\upnu =0.49$). Stiffer materials will only show a small deformation under loading, meaning that even for high values of $\nu$ the low strain of the mask material will not exert a substantial stress onto the skin surface. For highly compliant materials this effect is also minimal; whilst in this case the strains may be large, the modulus is low and therefore the resulting stress introduced into the interface will be too low to substantially affect the SED in the skin. These results, however, illustrate a potential issue for the typical softer materials used in respirator masks, which are often rubbery materials with a Young’s modulus that falls in the “intermediate” range. This means that the use of these materials in PPE may possibly need further consideration.

### Friction in the interface

A high interfacial friction coefficient between mask and skin results in increased levels of SED close to the skin surface. This is in agreement with literature, where high friction has been related to the development of superficial tissue injury^6,17,33^, whilst deep tissue injury appears to be related to the direct application of pressure^17^. In addition, friction may cause delamination of the dermal–epidermal junction, resulting in blisters and skin tearing ^35–38^. Therefore, reducing the level of friction should be of primary concern when designing respirator masks. It is worth noting that friction in the skin-PPE interface is not a parameter that can easily be designed or optimised. The overall friction between the skin and PPE is a system parameter that depends on the mask material, the characteristics of the skin, as well as the loading and interfacial conditions. However, the literature mentions various ways to reduce friction, including the use of custom surface microgeometry or textures on the respirator masks, controlling the moisture in the contact and the use of specialised lubricants^2,39,40^. The application of a (micro-)texture to the surface of a device may be an effective measure to reduce the shear forces ^41–43^. However, this may interfere with the sealing capability of the respirator.

Following mask usage, moisture levels in the skin are reported to increase ^44^. The frictional response of skin is strongly dependent on humidity ^45–47^ and a moist environment macerates the skin and locally disrupts the skin barrier function ^16,48^. Therefore, moisture control is an effective means of reducing friction and preventing injury. Breathable materials could be utilised, and inspiration may be drawn from the materials used in diapers and sanitary towels, both of which make contact with skin for extended periods of time in warm, humid conditions. An additional solution that may be considered, particularly by users that suffer from high friction or ‘sticky’ skin, is the use of a topical creams to alleviate the shear stresses in the skin-PPE interface^39,40^.

### Design considerations

**Figure 4:**
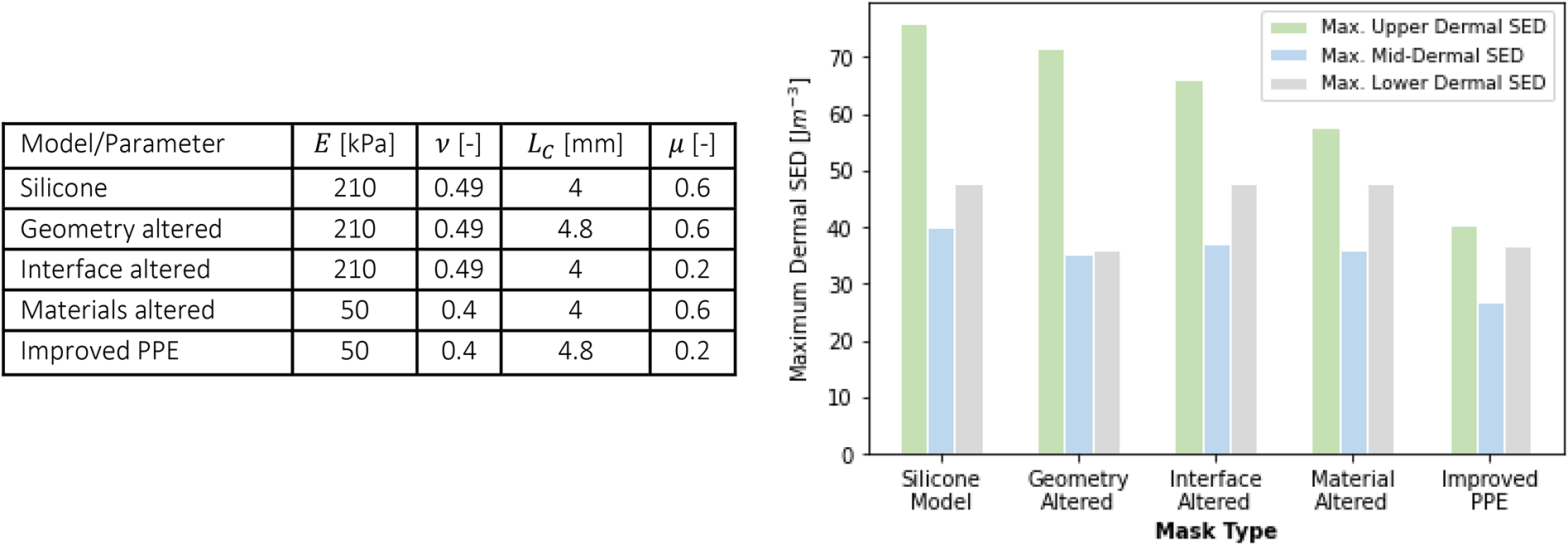
Table (left) showing different mask modifications compared to a silicone-based model. Graph (right) showing the SED in the upper (green), middle (blue) and lower dermis (grey) in response to the modifications.

The results presented provide insight into the relative importance of the various investigated parameters. These results can be used to extract design guidelines for facial PPE. Figure 4 shows the potential reduction in skin loading resulting from the use of alternative geometrical, interfacial and material parameters, taking a silicone-based face mask as the starting point^49^. The results confirm that interfacial and material alterations have a substantial effect on skin loading near the surface, whilst geometric alterations mainly effected the subsurface response.

Figure 4 also presents results obtained for an improved PPE, comprising an alternative material with increased contact area and reduced friction. The SED levels in the upper dermis reduce for every single alteration, resulting in an overall reduction of 46.6% for the improved model. The SED levels deeper in the tissue can only be reduced by changing the geometry of the mask. This enables the targeted augmentation of PPE to reduce the likelihood of injuries at specific dermal locations, deep tissue injury requires geometrical changes, whilst superficial injury may be alleviated through interfacial and material interventions.

Elastomeric materials are often used in respirator masks as the contacting layer against the skin. The low modulus of these materials distributes the pressure over a larger area, providing a degree of comfort whilst permitting a tight seal to form around the face to prevent leakage and viral exposure. However, the combination of the stiffness ($E<1 \mathrm{MPa}$) and near incompressibility ($\nu \to 0.5$) of these materials may result in an elevated SED in the skin, as shown in Fig. 2b. More significantly, in contact against skin, elastomers exhibit friction coefficients that exceed 1^39,40,50^ which significantly elevates dermal SED values. In terms of defining an optimal material to be used for facial PPE, the three parameters to consider are:

- a modulus of about 100 kPa. Elastomers with a lower modulus would be beneficial from a pressure point of view, but are well known for their adhesive behaviour, which would elevate shear stresses in the skin
- a Poisson’s ratio below 0.45 to reduce compression-induced shear stress
- a coefficient of friction against skin of approximately 0.2, but definitely not exceeding 0.5.

Whilst a single material with these properties may not be available, such a combination of characteristics may be achieved by using a soft, compressible inner layer (such as polymer foams or gels) covered with a thin, low-friction, breathable outer layer. Such a solution would confer beneficial bulk and interfacial properties to the user, reducing the risk of discomfort and injury.

### Strengths and limitations to the study

The objective of the developed model was to record the strain energy density in the dermis when in contact with PPE with a variety of properties, in order to indicate the propensity for tissue failure. The accuracy of the obtained results depends on how representative the model and input parameters are. Whilst geometric data is available for facial tissue, there is a lack of data accurately describing its topology and mechanical properties. Consequently, a generalised skin model was developed, comprising a smooth surface and typical geometric and mechanical properties. The effects of these assumptions on the results are limited; skin topography would result in locally elevated contact pressures on the epidermis, but the high stiffness of the epidermis prevents these pressures to be translated into elevated SED values into the subsurface tissue. Changing the geometry will affect the absolute values obtained, but not the trends observed. The mechanical properties of facial skin as reported in literature^51^ and used in this model are linearly elastic, whilst this is sufficient for the purposes of this study, any further optimisation will require a better understanding of the nonlinearity and time-dependent behaviour of each layer of the skin.

Additionally, the properties of facial tissues and craniofacial dimensions differ significantly between ages, ethnicities and sexes^52,53^ and therefore further work is required to enable differentiation and optimization of PPE design for different demographics in order to ensure functionality and fitting whilst preventing injury and viral exposure^54^. Finally, damage thresholds for skin have not yet been established and these would provide useful insight into the likelihood of failure at different facial locations.

## Materials and methods

### Simulating and varying skin–PPE contact

**Figure 5:**
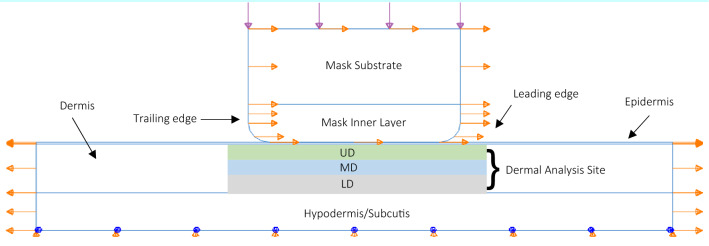
Schematic diagram detailing the compression and translation of a mask model against a skin model, in order to simulate PPE- Skin contact.

Figure 5 shows a schematic diagram of the developed finite element model which comprises the contact between a respirator mask and facial skin tissue. A two-dimensional, isotropic finite element models was developed using ABAQUS CAE 2019. The mask model was loaded with a uniform pressure of 1 N/mm along the 5 mm upper surface of the mask, thereby generating a 5 N applied load which was held constant throughout the study. This force caused the mask to indent the skin and generate a maximum pressure peak of 9.3 kPa at the edge of the mask (region ${P}_{L}$ in Fig. 1), similar to pressures reported in literature ^29,55–57^. The mask model was then translated by 2 mm to represent the relative motion of facial PPE whilst executing day-to-day tasks. As the exact damage threshold for skin has not been established and will vary interpersonally, the main purpose of this numerical investigation is to establish the effects and trends that a range of mask modifications have on the resulting subsurface SED distribution in the skin tissue, rather than attempting to obtain absolute values. The obtained results enable the recommendation of a range of potential mask modifications and help identifying the likely sites of skin failure. Initial simulations involved independently varying the elastic modulus, Poisson’s ratio, contact length and interfacial friction coefficient of the mask whilst holding the other parameters constant at a reference value, as listed in Table 1. The SED values in the tissue were recorded in order investigate the effects of these modified material, geometric and interfacial properties of the mask on the damage propensity of the skin. Subsequently, based on the obtained initial results, an improved PPE comprising the combined effects of a modified geometry, material and interface, was proposed and its effects on the skin SED values were investigated (Fig. 4).

### Skin model development

The skin model consists of three layers, the epidermis, dermis and hypodermis or subcutaneous tissue, which were modelled as a continuum with flat interfaces between them. The model was developed as a single geometry before being partitioned into individual sections with different thicknesses and assigned material properties, thus simulating perfect adhesion between the skin layers. The skin comprises an epidermal layer with a thickness of 0.05 mm on top of a 1.3 mm thick dermal layer. This system is supported by a 1 mm subcutaneous tissue layer that provides a compliant boundary condition for the dermis by representing underlying tissue. The model was given material and geometric properties from literature^58–61^, listed in Table 2. Data on facial skin was used where available, complemented with properties of volar forearm skin, as research suggests there is no significant difference in thicknesses, stiffness, Poisson’s ratios and frictional behaviour^62,63^. The modelled section of skin has a width of 15 mm, of which a 6 mm wide section is designated as the dermal analysis site. The additional width of the skin model will mitigate any edge effects on the dermal analysis site and allows sufficient room at the surface for translation of the PPE. The skin model comprised 40,014 quadratic plane strain elements.

**Table 2:** Material and geometric parameters of the simulated skin model.

Skin layer	E (kPa)^58^	$\nu$ (–)^61^	Thickness (mm)^59,60^
Epidermis	1500	0.48	0.05
Dermis	20	0.48	1.3
Hypodermis/subcutis	2	0.48	1.0

As a form-fitting external barrier for the human body, the skin in its natural state is subject to tensile stresses. In order to keep the model of the skin taut, a pre-tensioning horizontal displacement of 0.1 mm was applied as a boundary condition on the left and right side of the finite element model. The base of the subcutis was restricted from vertical and rotational motion. Literature suggests adhesive friction is the main cause of macroscopic friction for unlubricated skin contacts^64^. Therefore, the surfaces were kept geometrically smooth, thus maximising the area of contact and consequently the adhesive friction acting on the interface. In the reference situation an interfacial coefficient of friction of 0.5 was applied between the mask and skin models. Following the translation of the skin, data was extracted from the dermal analysis site, underneath the mask-skin contact area. To reduce mesh effects, the reported maximum SED value is the average of the five elements with the highest SED values. Values are reported for the total skin, but also partitioned into an upper dermal segment (UD) and a mid-dermal segment (MD) both with a thickness of 0.4 mm and a lower dermal segment (LD) of 0.5 mm. These dimensions were chosen purely for analysis purposes and to provide insight, and do not refer to any specific anatomical dermal sublayers.

### Mask model development

The mask model represents the edge or rim of a respirator mask which is in contact with cheek skin. The mask comprises two components, a 2 mm thick substrate which is exposed to the environment, and an inner layer which is in contact with skin and for which the properties are varied in this study. The total width of the mask is 5 mm and the substrate material is modelled with an elastic modulus of 7 MPa and a Poisson’s ratio of 0.4. The inner layer of the mask is perfectly adhered to this substrate and has a thickness of 1 mm. The edges were given a radius of curvature of 0.5 mm, which was varied later in the study to adjust the area of contact between the skin and the PPE. The mask model was given a coarser mesh than the skin, consisting of 971 quadratic plane strain elements.
